# Metabolomics revealed pharmacodynamic effects of aspirin and indobufen in patients after percutaneous transluminal angioplasty surgery

**DOI:** 10.3389/fcvm.2024.1433643

**Published:** 2024-10-29

**Authors:** Shaobo Sun, Kang Xun, Damei Li, Renjie Bao

**Affiliations:** Department of Nephrology, The People’s Hospital of Suzhou New District, Suzhou, Jiangsu, China

**Keywords:** vascular restenosis, aspirin, indobufen, plasma, metabolomics, amino acids, biomarker

## Abstract

**Introduction:**

Aspirin and indobufen are commonly used therapeutic drugs for the prevention of vascular restenosis (VR) after percutaneous transluminal angioplasty surgery. They both exhibited antiplatelet effects but molecular mechanisms underlying metabolic changes induced by them remain unclear.

**Methods:**

In this study, we collected plasma samples from patients on aspirin medication (*n* = 5), patients on indobufen medication, patients with no medication after PTA, and healthy controls (CKs) (*n* = 5). Our investigation aimed to reveal the metabolic processes in patients during vascular restenosis and its amelioration through drug therapy using liquid chromatography-tandem mass spectrometry (LC-MS/MS).

**Results:**

Our data showed significant alterations in amino acid and choline metabolism in patients without medication after PTA. Aspirin and indobufen were able to regulate these metabolic pathways to alleviate VR symptoms. We identified several characteristic amino acids, including pro-leu, L-citrulline, his-glu, and L-glutamate, as important biomarkers for VR assessment in patients without medication after PTA. A total of 17 and 4 metabolites involved in arginine and phenylalanine metabolism were specifically induced by aspirin and indobufen, respectively. Their expression levels were significantly regulated by aspirin or indobufen, nearly reaching normal levels.

**Discussion:**

Taken together, our identification of metabolites involved in metabolic changes affected by aspirin and indobufen medication enhances the understanding of VR pathology after PTA. This may help identify early diagnostic biomarkers and therapeutic targets

## Introduction

Chronic kidney disease (CKD) poses a major public health challenge, leading to high morbidity and mortality among the elderly worldwide (1). The prevalence of CKD progressing to end-stage kidney disease (ESKD) is increasing due to complications such as obesity, diabetes, and hypertension, necessitating lifelong hemodialysis and medication (1, 2). Statistically, the number of ESKD patients has reached 250 million worldwide, imposing significant economic and social burdens on individuals, families, and countries (3).

Autogenous arteriovenous fistula (AVF) is a widely used hemodialysis method for treating ESKD patients, offering high safety and long-term usability (4). However, prolonged usage can lead to vascular stenosis in patients. Notably, vascular stenosis caused by AVF is typically treated surgically, but surgery often results in significant wastage of vascular resources. With the development of interventional techniques, percutaneous transluminal angioplasty (PTA) has been widely adopted in clinical practice (5, 6). Due to its minimally invasive nature, safety, rapid postoperative recovery, and the ability for patients to commence AVF use sooner, PTA has gradually become the preferred treatment for vascular stenosis in AVF patients (5, 7). Unfortunately, within the first six months or more following percutaneous transluminal angioplasty (PTA), patients may experience recurrence of stenosis or acute vascular occlusion in their arteriovenous fistula (AVF) (6). Although PTA can be repeated for recurrent cases, it may affect the longevity of AVF use and lacks awareness of recurrent stenosis events (7).

Although the pathological mechanism underlying the stenosis remains uncertain, recent documents illustrated that it is a kind of repair reaction caused by multiple growth and cytoplasmic factors after vascular injury, leading to intimal hyperplasia (8). Mechanistically, normal vascular smooth muscle cells (VSMCs) in the vascular medium undergo phenotypic transformation in response to the injury of endothelial cells in the medial wall of the blood vessel, from the physiological state of contraction to synthesis. Synthetic VSMCs migrate to the inner membrane, where they proliferate and secrete a large extracellular matrix (ECM) to repair damaged blood vessels (9). On the other hand, various growth factors and cytokines released from platelets, such as platelet-derived growth factor (PDGF), fibroblast growth factor (FGF), interleukin 1 (IL-1), and tumor necrosis factor alpha (TNF-α), invade macrophages or activated VSMCs, further causing VSMCs to proliferate and migrate (9, 10). Proliferating VSMCs secrete ECM, leading to endothelial hyperplasia and vascular remodeling, resulting in restenosis. Therefore, inhibiting the phenotypic transformation of VSMCs, reducing the abnormal proliferation and migration of VSMCs may effectively prevent restenosis after PTA.

Given that endothelial cell injury caused by interventional therapy can promote platelet agglutination, activate coagulation factors, thrombin, etc., thereby accelerating the formation of acute thrombus shortly after PTA (11). In current clinical practice, adjunctive use of drugs with dual anti-coagulant and anti-platelet aggregation effects, such as aspirin and indobufen, has been shown to be indispensable for prevention of post-PTA restenosis in patients (12, 13). Both medications function by inhibiting cyclooxygenase-1 (COX-1) activity, reducing thromboxane A2 (TXA2) synthesis, and preventing thrombotic or vascular occlusive events (13). However, aspirin acetylates the serine residue of COX, blocking the binding of COX catalytic site with substrates, leading to permanent COX inactivation and inhibition of platelet activity until platelet regeneration occurs. In contrast, the inhibitory effect of indobufen on platelets is reversible (12, 14). Additionally, it reduces platelet aggregation by inhibiting adenosine diphosphate (ADP), adrenaline, and other platelet activation factors, shortening the time to reach peak levels in the body and effectively reducing adverse reactions in patients (14). Long-term aspirin use is associated with bleeding complications, gastrointestinal irritation symptoms, diarrhea, rash, and other adverse effects (15). Therefore, indobufen is typically used as an alternative treatment for patients with a history of gastrointestinal bleeding or intolerance. Despite the widespread clinical use of aspirin and indobufen, understanding the molecular pathogenesis of post-PTA restenosis remains limited. Moreover, in clinical practice, it is necessary to assess the effectiveness and safety of drugs for different types of CKD patients and seek reliable biomarkers for preventive diagnosis during drug use. This will enrich our understanding of their pharmacological mechanisms and help prevent adverse drug reactions.

With the rapid development of mass spectrometry (MS) offering high resolution and fast scanning metabolomics has emerged as an indispensable analytical tool to study changes in metabolite abundances at the cellular level under various physiological or pathological conditions (16). The analysis of global metabolite abundances from serum, plasma, and other tissues can uncover characteristic metabolite expressions for effective diagnosis and therapy (17). However, the changes in metabolites occurring in vascular restenosis after PTA remain poorly understood. In this study, we collected plasma samples from patients with restenosis who were treated with either aspirin or indobufen. We conducted a comparative metabolomic analysis to elucidate global metabolite changes among patients receiving different therapeutic methods using liquid chromatography-mass spectrometry (LC-MS). Our work may provide valuable guidance for better understanding the pathogenesis of restenosis and facilitate the identification of new potential biomarkers or therapeutic targets for this condition.

## Results

### Aspirin and indobufen exhibit alleviative effects to vascular restenosis after PTA

A total of 20 plasma samples were obtained from 10 male patients with vascular stenosis who underwent aspirin (PTA_A) or indobufen (PTA_INDO) therapy, 5 patients who did not take any medication after percutaneous transluminal angioplasty (PTA_UN), and 5 healthy individuals as controls (CK). Eight clinical characteristics were examined in each group: age, creatinine, triglyceride, low-density lipoprotein cholesterol (LDL-C), high-density lipoprotein cholesterol (HDL-C), hemoglobin, C-reactive protein (CRP), and D-dimer. The levels of creatinine, triglyceride, LDL-C, CRP, and D-dimer were significantly elevated in the PTA_UN patient group compared to healthy individuals, suggesting a high risk for kidney disease, hyperlipidemia, cardiovascular disorder, and thrombosis. We noticed that high level of these clinical indexes indicated a stress response after PTA, and which were markedly improved by aspirin and indobufen therapy, demonstrating medications of aspirin and indobufen could alleviate this response and prevent thrombosis (Table 1). Meanwhile, creatinine and LDL-C were positively related with proliferation and migration of VSMCs, illustrating that aspirin and inodubfen could suppress the activity of VSMCs (Table 1).

**Table 1:** Demographic and clinical characteristics of the study population.

Item	CK	PTA_UN	PTA_A	PTA_Indo
Age (years)	60.14 ± 3.17	62.83 ± 2.38	62.16 ± 4.03	59.46 ± 3.71
Creatinine (*μ*mol/L)	135.81 ± 33.8a	1,142.51 ± 116.62b	813.93 ± 90.91c	627.15 ± 100.34d
Triglyceride (mmol/L)	1.62 ± 0.05a	2.26 ± 0.03b	1.20 ± 0.09c	1.37 ± 0.05c
LDL-C (mmol/L)	1.87 ± 0.06a	3.89 ± 0.67b	2.73 ± 0.53c	2.62 ± 0.45c
HDL-C (mmol/L)	1.27 ± 0.08	1.046 ± 0.07	1.11 ± 0.05	1.07 ± 0.07
hemoglobin (g/L)	142.31 ± 7.34	109.20 ± 21.34	114.13 ± 14.18	96.16 ± 30.18
CRP (mg/L)	2.64 ± 1.91a	4.74 ± 1.05b	3.65 ± 0.54c	2.97 ± 1.71c
D-dimer (mg/L)	0.22 ± 0.06a	1.56 ± 0.45b	0.56 ± 0.19c	0.75 ± 0.39c

The clinical data was presented as mean ± SD. ANOVA analysis was used to compare the difference among four groups. CK represented healthy controls. LDL-C, low-density density lipoprotein cholesterol; HDL-C, high-density lipoprotein cholesterol; CRP, C-reactive protein.

Different lowercase letters indicated significant difference with *P* < 0.05.

**Figure 1:**
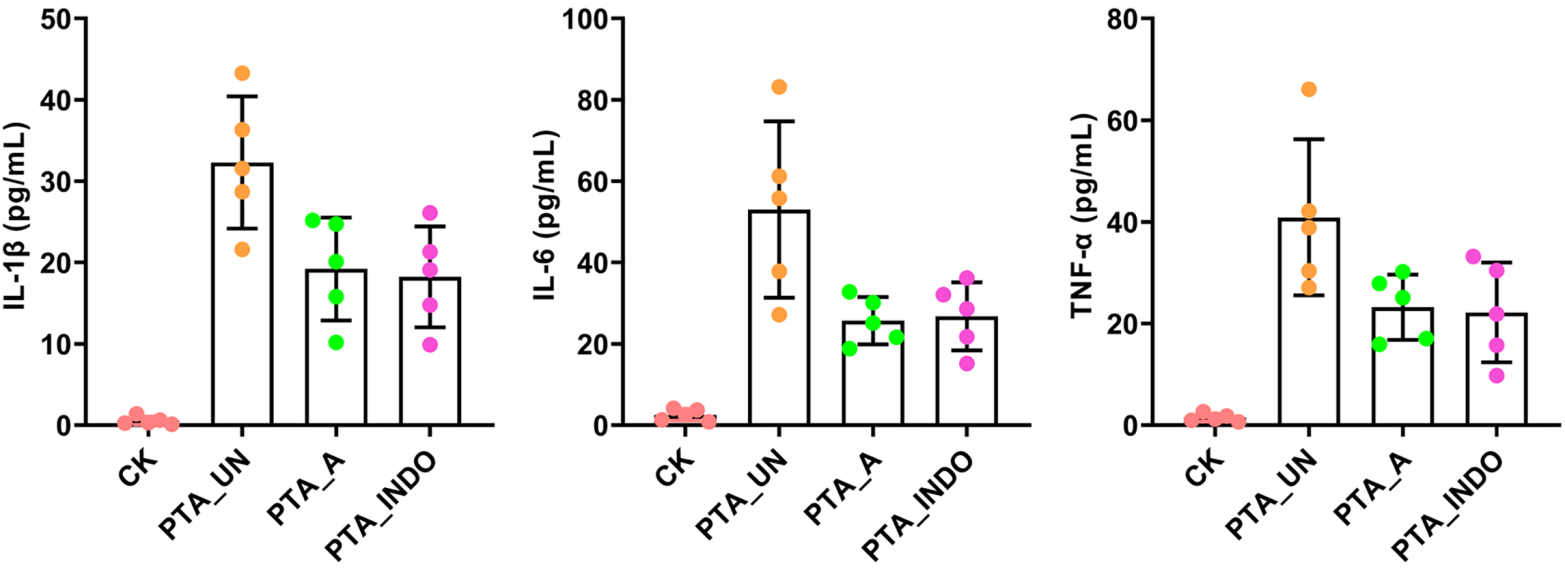
ELISA validation of IL-1β, IL-6 and TNF-α in plasma under PTA surgery. Aspirin and indobufen significantly attenuated expression level of inflammatory factors in plasma after PTA surgery. Different lowercase letters indicated significant difference by one-way ANOVA with Tukey's HSD test (*P* < 0.05).

Absolutely, vascular endothelial injury is inevitable in PTA surgery, we also examined some inflammation-related factors, including interleukin-1β (IL-1β), IL-6 and tumor necrosis factor-α (TNF-α) in patient plasma by enzyme-linked immunosorbent assay (ELISA). The results showed that they were significantly up-regulated after PTA surgery, while their concentration was reduced by adjuvant utilization of aspirin and indobufen medications after PTA, demonstrating contributions of aspirin and indobufen in the reduced risk of vascular restenosis (Figure 1). Notably, we found that indobufen exhibited better therapy effects to reduction of stenosis-related factors (Table 1 and Figure 1), to further investigate the difference in therapy effects, we delved into deciphering specific metabolic changes induced by aspirin and indobufen. Plasma samples from these patients and healthy individuals were analyzed using liquid chromatography-mass spectrometry (LC-MS).

### PTA surgery significantly altered amino acid and choline metabolisms in patients

**Figure 2:**
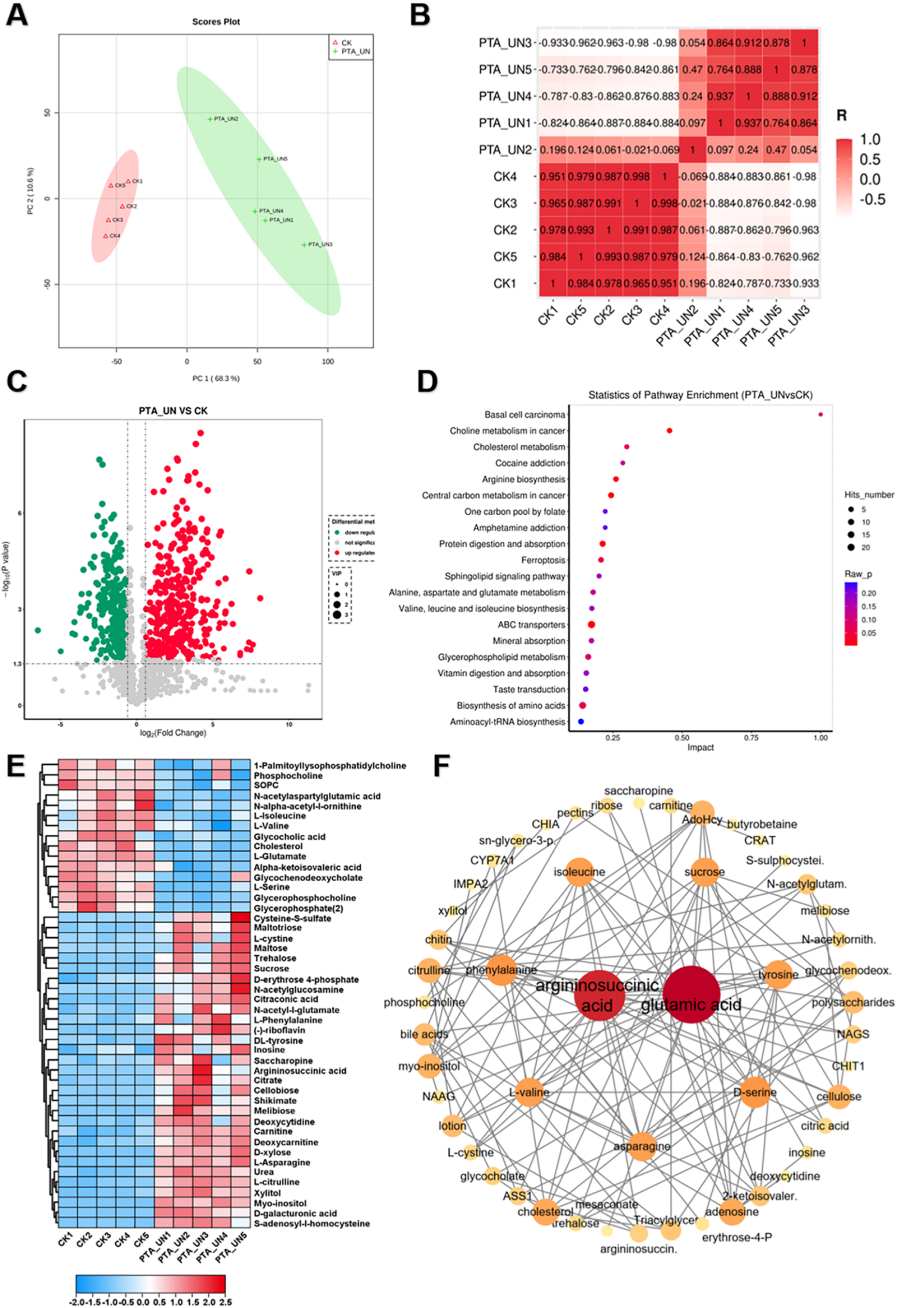
A global view of metabolomic changes altered by percutaneous transluminal angioplasty (PTA) surgery. **(A)** Principal component analysis (PCA) exhibited the distribution of metabolome profiles between PTA_UN and CK. **(B)** Pearson correlation analysis between samples from PTA_UN and CK. **(C)** Volcano plot illustrated the differentially expressed metabolites (DEMs) satisfying the screening threshold of │log_2_(foldchange)│>1 and VIP >1.0 in the comparison of PTA_UN vs. CK. **(D)** Kyoto Encyclopedia of Genes and Genomes (KEGG) enrichment analysis showed the functional distribution of DEMs. **(E)** Heatmap exhibited the expression pattern of metabolites involved in amino acids and choline metabolism pathways in PTA_UN vs. CK. **(F)** The construction of interaction network of metabolites depicted in heatmap. The circle size means importance of metabolite in the network. Hub metabolites were reflected by universal correlation with other metabolites.

To investigate the metabolome changes induced by PTA surgery, a pairwise comparison of metabolome profiles between PTA_UN and CK was conducted. As shown in Figure 2A, principal component analysis (PCA) revealed a clear separation of metabolome profiles between PTA_UN and CK, with variations of PC1 (68.3%) and PC2 (10.6%). This suggested that PTA alters metabolome patterns, providing reliable profiles for further study. Consistently, Pearson correlation analysis showed close aggregation of metabolome profiles within each group, supporting the PCA results (Figure 2B). Therefore, PTA surgery significantly affected the metabolome patterns in the patient group compared to healthy individuals. Furthermore, the differentially expressed metabolites (DEMs) satisfying the screening threshold of VIP >1.0 and │log_2_(foldchange) │>1 were depicted in a volcano plot in PTA_UN vs. CK. A total of 669 DEMs was identified, including 377 up-regulated and 292 down-regulated metabolites (Figure 2C). These DEMs were further mapped using Kyoto Encyclopedia of Genes and Genomes (KEGG) enrichment analysis to explore their potential molecular functions. As depicted in Figure 2D, DEMs were mainly enriched in pathways such as choline metabolism, arginine biosynthesis, central carbon metabolism, ABC transporters and amino acids biosynthesis. And then, metabolites involved in these pathways were further represented in a heatmap to show their expression in the comparison between PTA_UN and CK. Notably, we observed that metabolites associated with amino acid and choline metabolism were the most significantly altered pathways by PTA, especially argininosuccinic acid, L-asparagine and L-citrulline with significant up-regulation as well as 1-palmitoyllysophosphatidyl choline, phosphocholine and L-glutamate with sharp decrease in PTA_UN group (Figure 2E). These metabolites are known to be essential biomarkers for multiple disease diagnoses and treatment indices (18). Importantly, increasing evidence have shown that increased accumulation of argininosuccinic acid and L-Citrullin contributed to promote production of NO, thus suppressing proliferation of VSMCs (9, 19), while 1-Palmitoyllysophosphatidyl Choline and phosphocholine were closely associated with inflammatory responses and promote the proliferation and migration of VSMCs (20). Meanwhile, metabolites in heatmap were linked into STITCH (Search Tool for Interacting Chemicals) online program to explore interactions of chemicals. As shown in Figure 2F, amino acids, especially for argininosuccinic acid and glutamic acid, were hub metabolites network showing their universal interactions in this interaction, demonstrating their changes in expression profoundly affected plasma metabolic process after PTA surgery, which deserved to be monitored in disease progression.

### Aspirin administration significantly induced metabolic changes related to arginine metabolism after PTA surgery

**Figure 3:**
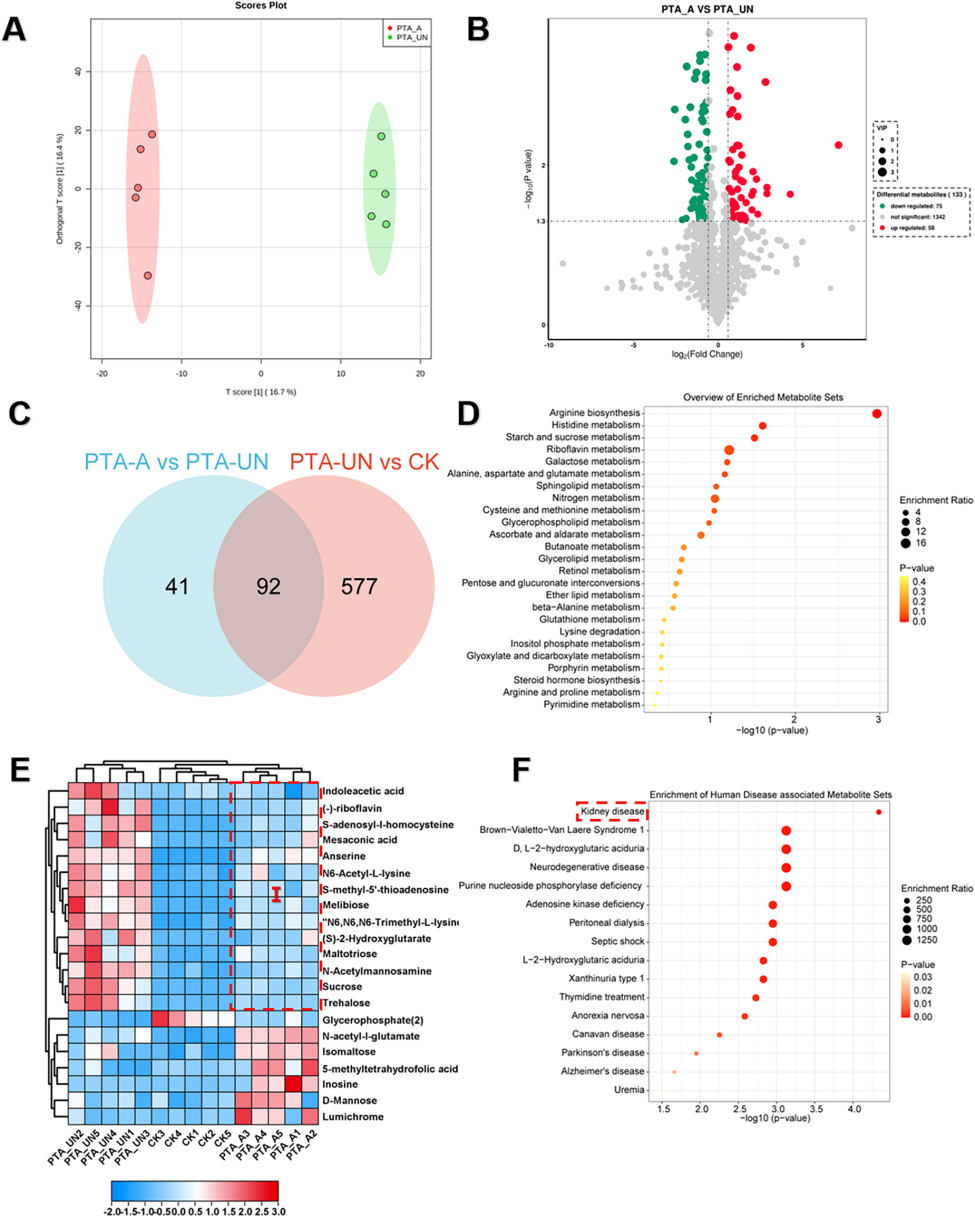
Aspirin administration significantly induced metabolic changes relevant to arginine metabolism compared to PTA patients with undisposed. **(A)** Orthogonal partial least-squares discrimination analysis (OPLS-DA) exhibited the variations of metabolome profiles between PTA_A and PTA_UN. **(B)** Volcano plot illustrated the differentially expressed metabolites (DEMs) satisfying the screening threshold of │log_2_(foldchange)│>1 and VIP >1.0 in the comparison of PTA_A vs. PTA_UN. **(C)** Venn diagram counted the numbers of metabolites shared in PTA_A vs. PTA_UN and PTA_UN vs. CK. **(D)** Kyoto Encyclopedia of Genes and Genomes (KEGG) enrichment analysis showed the functional distribution of DEMs identified in Venn plot. **(E)** Heatmap exhibited the expression pattern of metabolites involved in enrichment analysis. **(F)** Enrichment analysis of metabolites associated with human disease. Metabolites in group I with red dotted line in **(E)** were further enriched in **(F)**.

To explore aspirin-induced metabolic changes, we performed a pairwise comparison of PTA_A vs. PTA_UN. A supervised orthogonal partial least-squares discrimination analysis (OPLS-DA) was constructed to evaluate the reliability of the tested model (Figure 3A). The results confirmed a 16.7% variation in PC1 between PTA_A and PTA_UN, suggesting that a portion of metabolites were markedly affected by PTA_A compared to PTA_UN. Based on this model, we identified 133 DEMs, including 58 up-regulated and 75 down-regulated metabolites (Figure 3B). Furthermore, we compared the metabolites involved in PTA_A vs. PTA_UN and PTA_UN vs. CK, showing 92 shared metabolites in both groups, supposing that expression of these metabolites may be influenced by aspirin administration (Figure 3C). We then linked them to KEGG enrichment analysis, showing they were mainly enriched in arginine biosynthesis and riboflavin metabolism (Figure 3D). Expression analysis confirmed that aspirin mainly up-regulated the expression of metabolites related to arginine metabolism. Importantly, we noticed that metabolites in group I were significantly up-regulated after PTA surgery compared to healthy individuals, and their expression was partially suppressed by PTA with aspirin administration (Figure 3E), suggesting that aspirin administration adjusted metabolite expression closer to normal levels. Notably, highly accumulated S-adenosyl-l-homocysteine and insufficient 5-methyltetrahydrofolic acid probably increased the risk of vascular restenosis by triggering the proliferation and migration of VSMCs (21, 22), and our data showed aspirin significantly improved the conditions by regulating their expression (Figure 3E). Further enrichment analysis illustrated that these metabolites showed a significant association with kidney disease (Figure 3F), highlighting their importance and potential values for biomarkers in kidney disease. Overall, these findings demonstrated that aspirin medication may improve arginine metabolism in plasma of patients with PTA for preventing vascular restenosis in disease progression.

### Indobufen administration improved phospholipid and immune related metabolism compared to PTA patients with undisposed

**Figure 4:**
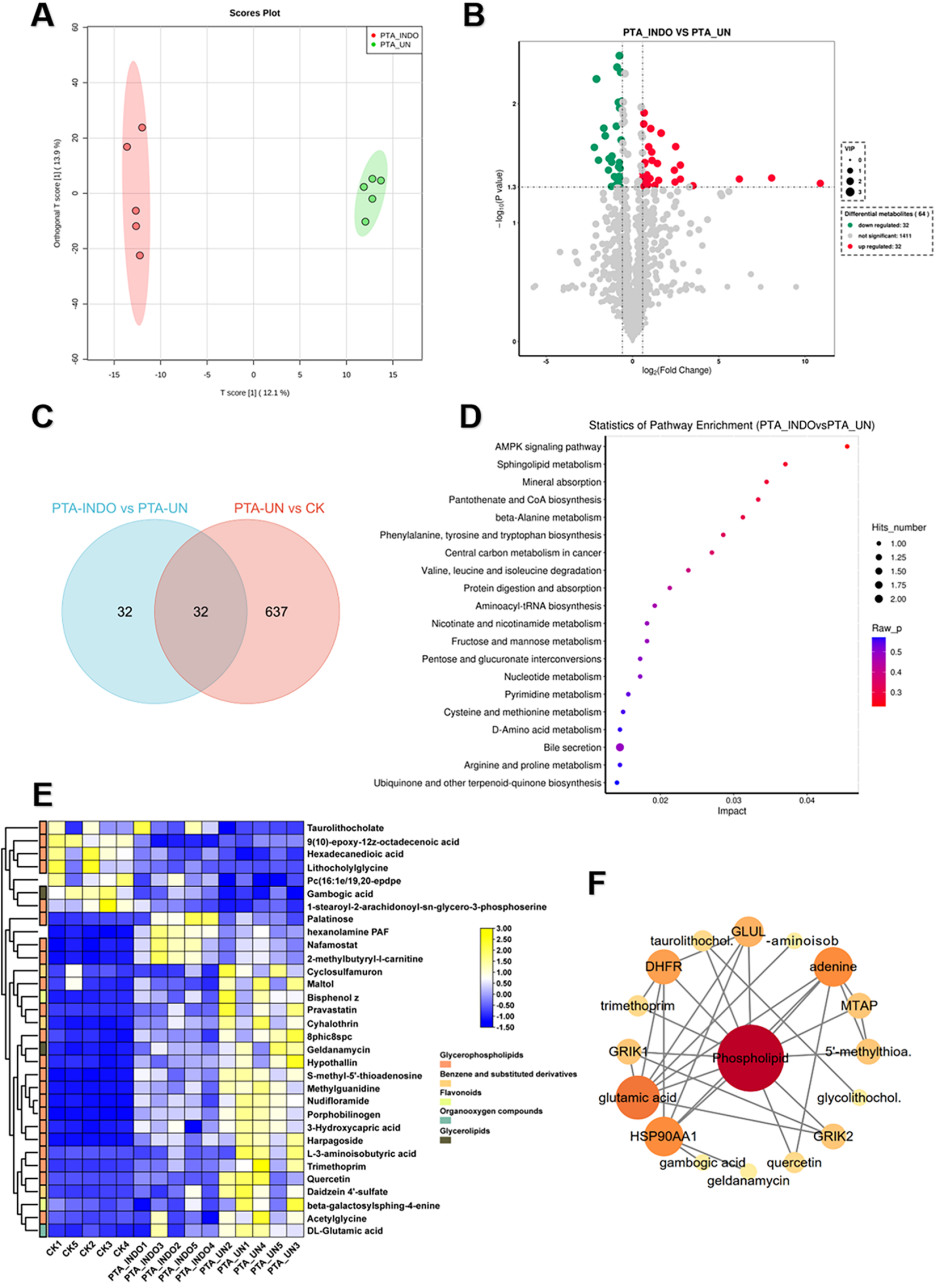
Indobufen administration significantly induced metabolic changes relevant to phospholipid and immune related metabolism compared to PTA patients with undisposed. **(A)** Orthogonal partial least-squares discrimination analysis (OPLS-DA) exhibited the variations of metabolome profiles between PTA_INDO and PTA_UN. **(B)** Volcano plot illustrated the differentially expressed metabolites (DEMs) satisfying the screening threshold of │log_2_(foldchange)│>1 and VIP >1.0 in the comparison of PTA_INDO vs. PTA_UN. **(C)** Top20 metabolites illustration according to VIP scores in PTA_INDO vs. PTA_UN. **(D)** Kyoto Encyclopedia of Genes and Genomes (KEGG) enrichment analysis showed the functional distribution of DEMs identified in volcano plot. **(E)** Venn diagram counted the numbers of metabolites shared in PTA_INDO vs. PTA_UN and PTA_UN vs. CK. **(F)** Heatmap exhibited the expression pattern of metabolites involved in enrichment analysis in PTA_INDO vs. PTA_UN.

To explore indobufen-induced metabolic changes, we developed a pairwise comparison of PTA_INDO vs. PTA_UN. OPLS-DA analysis revealed different metabolome patterns between PTA_INDO and PTA_UN with a PC1 variation of 12.1% (Figure 4A), ensuring a stable testing model for further metabolite identification. Ultimately, 32 up-regulated DEMs and 32 down-regulated DEMs were identified in the comparison of PTA_INDO vs. PTA_UN (Figure 4B). We noticed that less number of metabolites were altered by indobufen medication compared to aspirin, only 32 metabolites were identified in comparison between PTA_INDO vs. PTA_UN and PTA_UN vs. CK (Figure 4C). Further KEGG enrichment analysis illustrated the functional distribution of above metabolites, showing that DEMs related to the AMPK signaling pathway, sphingolipid metabolism, mineral absorption, pantothenate and CoA biosynthesis, and beta-alanine metabolism were markedly aggregated (Figure 4D). These metabolites were further represented in heatmap showing expression of them in three groups. Expression analysis showed that indobufen mainly altered the expression of metabolites related to phospholipids (Figure 4E). Intriguingly, we found that two groups of metabolites closely associated with indobufen tended to sustain their levels closer to normal with indobufen treatment (Figure 4F). Additionally, we found that several metabolites were specifically induced by indobufen, such as palatinose, hexanolamine PAF, nafamostat, and 2-methylbutyryl-L-carnitine (Figure 4F). hexanolamine PAF is a phospholipid compound belonging to platelet-activating factor, which is known to be closely related to inflammatory responses, platelet aggregation, vascular smooth muscle cell proliferation and atherosclerosis (23). Meanwhile, it was reported that increase of 2-methylbutyryl-L-carnitine in the plasma of patients with steatohepatitis (NASH) could be used as one of the indicators for the diagnosis of metabolic diseases (24). This finding implied that we should consider whether indobufen administration is suitable for PTA patients with NASH. Meanwhile, metabolites with markedly expression were applied to conduct a potential interaction network, illustrating co-expression and regulation among them (Figure 4F). The result showed that phospholipid was hub metabolite in this network exhibiting strong correlations with glutamic acid, HSP90AA1, DHFR, GLUL, MTAP and GRIK2, which were considered as biomarkers or drug targets in multiple cancers and disease (25).

### Aspirin exerted more obvious effect on arginine metabolism than that of indobufen

**Figure 5:**
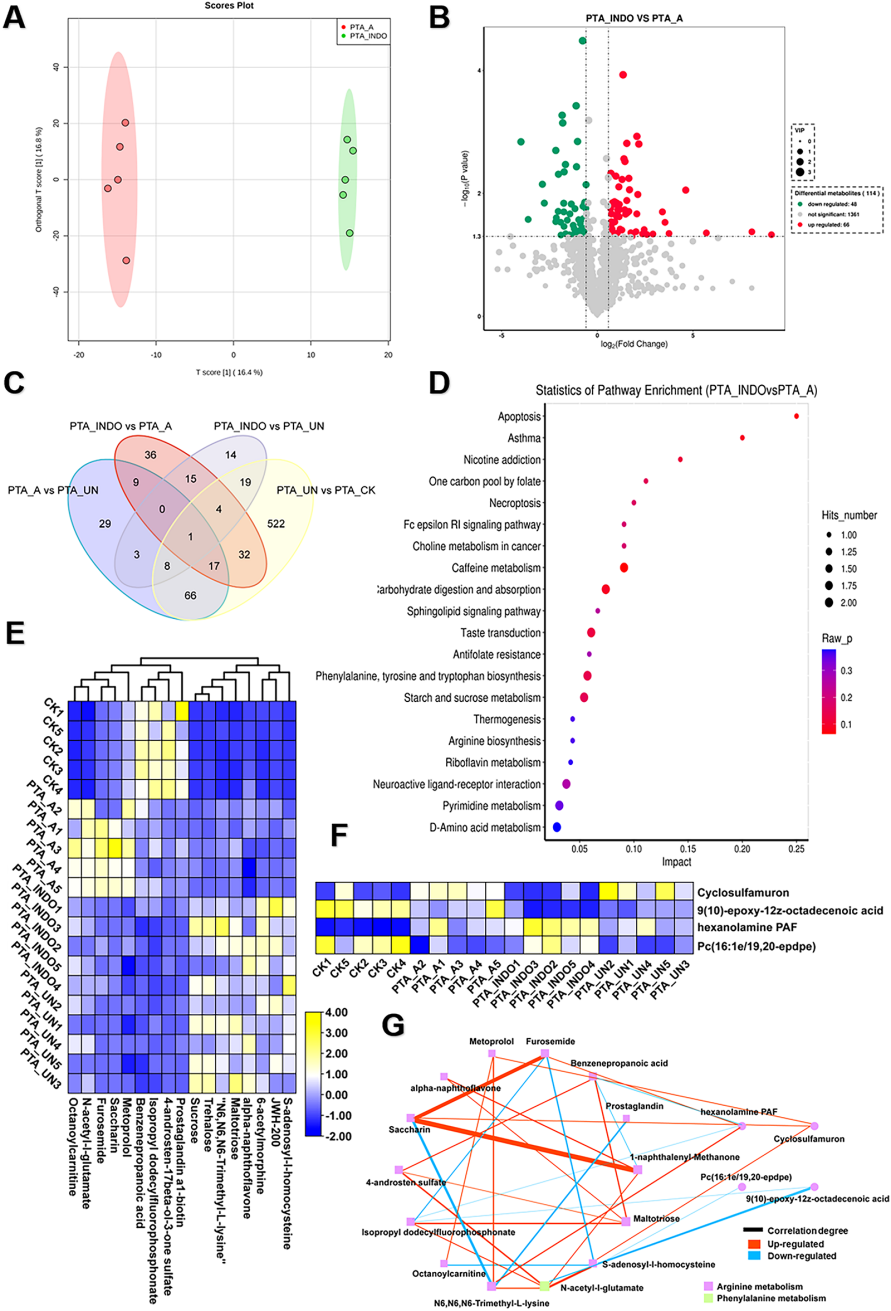
Aspirin administration significantly shaped metabolic changes relevant to arginine metabolism compared to PTA patients with indobufen. **(A)** orthogonal partial least-squares discrimination analysis (OPLS-DA) exhibited the variations of metabolome profiles between PTA_INDO and PTA_A. **(B)** Volcano plot illustrated the differentially expressed metabolites (DEMs) satisfying the screening threshold of │log_2_(foldchange)│>1 and VIP >1.0 in the comparison of PTA_INDO vs. PTA_A. **(C)** Kyoto Encyclopedia of Genes and Genomes (KEGG) enrichment analysis showed the functional distribution of DEMs identified in volcano plot. **(D)** Venn diagram counted the numbers of metabolites shared in PTA_INDO, PTA_A and PTA_UN and CK groups. **(E,F)**. Heatmap exhibited the expression pattern of metabolites that were specifically induced by indobufen and aspirin respectively in four groups. **(G)** Topological correlation network of metabolites associated with indobufen and aspirin administration.

To compare the metabolic influence shaped by aspirin or indobufen, the comparative metabolomic analysis of PTA_INDO vs. PTA_A was investigated. OPLS-DA analysis constructed a reliable model, showing that the 16.4% variation in metabolome patterns was significant for further metabolite determination (Figure 5A). A volcano plot illustrated that a total of 66 up-regulated DEMs and 48 down-regulated DEMs were identified in PTA_INDO vs. PTA_A (Figure 5B). The DEMs were subsequently mapped using KEGG enrichment analysis to determine their relative metabolic functions. We noted that these metabolites were significantly enriched in caffeine metabolism, carbohydrate digestion and absorption, taste transduction, phenylalanine, tyrosine, and tryptophan biosynthesis, and starch and sucrose metabolism (Figure 5C).

Furthermore, we performed Venn analysis to determine the specific metabolite clusters related to aspirin and indobufen. As shown in Figure 5D, 8 and 17 metabolites were specifically responsive to indobufen and aspirin, respectively. After data filtering, their expression patterns were illustrated in heatmaps (Figures 5E, F), showing the alterations of these metabolites as influenced by indobufen and aspirin. A detailed topological network was visualized using Cytoscape to investigate the importance of metabolites in the network (Figure 5G). We noticed that most metabolites were involved in the modulation of arginine and phenylalanine metabolism, suggesting that indobufen and aspirin induced significant metabolic changes by affecting arginine metabolism. In common, we found both medicines exhibited suppressed effects to govern the concentration of metabolites which were risk for eliciting progression of vascular restenosis or other concurrent disease. Meanwhile, furosemide, saccharin, and 1-naphthalenyl-methanone showed a strong tendency of correlation, suggesting their potential as biomarkers for monitoring the patient course with aspirin administration. N6, N6, N6-trimethyl-L-lysine, N-acetyl-L-glutamate, and hexanolamine PAF were imperative in the network and termed hub metabolites in response to aspirin and indobufen administration.

## Materials and methods

### The information of test population

A total of 15 male patients with vascular stenosis and 5 healthy male individuals were admitted to the Suzhou High-Tech Zone People's Hospital from March 20 to September 20, 2023. All patients received percutaneous transluminal angioplasty (PTA) surgery due to vascular stenosis. Among them, 5 patients did not take any medication after PTA (PTA_UN), while 5 patients took aspirin 100 mg twice a day, and 5 patients took indobufen 100 mg twice a day. Plasma samples from these patients were collected and sequenced three months after PTA. Aspirin and indobufen were purchased from Bayer Schering Pharmaceutical AG, Leverkusen, Germany (100 mg/tablet).

### Sample collection and processing

Fasting blood samples (5 mL) from patients and healthy individuals were drawn into BD vacutainer tubes (JSHXRT, China) in the morning. The plasma was separated by centrifugation at 3,000 g for 20 min at 4°C. Plasma samples in the supernatants were immediately dispensed into sterile centrifuge tubes (F609693#Sangong, China) and stored as aliquots at −80°C until processing.

### UHPLC-MS/MS analysis

All samples were acquired using the LC-MS system followed machine instructions. First, all chromatographic separations were performed using an ultra-performance liquid chromatography (UPLC) system (SCIEX, UK). ACQUITY UPLC T3 column (100 mm × 2.1 mm, 1.7 µm, Waters, UK) was used for the reversed-phase separation. The column oven was maintained at 50°C. The flow rate was 0.3 mL/min and the mobile phase consisted of solvent A (0.1% formic acid in water) and solvent B (0.1% formic acid in ACN). Gradient elution conditions were set as follows: 0–0.5 min, 5% B; 0.5–2.5 min, 5%–70% B; 2.5–7.5 min, 70%–100% B; 7.5–9.0 min, 100%, 9.0–9.5 min, 100%–5%; 9.5–12 min, 5% B. Sample metabolic analytes flowing from the column were collected in positive and negative mode by high-resolution mass spectrometry Triple TOF 5,600 + . The detailed parameters are as follows: Ion Source Gas1: 50 psi, Ion Source Gas2: 50 psi, Curtain Gas: 35 psi, Source Temperature: 500°C, IonSapary Voltage Floating: 5,500 V & −4,500 V (positive & negative); Declustering Potential (DP): ±80 V (positive & negative); TOF MS scan m/z range: 60–1,200 Da, Product ion scan m/z range: 25–1,200 Da, TOF MS scan accumulation time 0.25s/spectra, Product ion scan accumulation time 0.03 s/spectra; The secondary mass spectrometry was obtained using Information Dependent Acquisition (IDA) and was in High Sensitivity mode, CE: 30V ± 15.

### Data processing and analysis

The raw data were converted to the mzML format using ProteoWizard and processed with an in-house program, which was developed using R and based on XCMS, for peak detection, extraction, alignment and integration. Then internal MS2 databases (Allwegene.DB) were used for metabolite annotation. metabolite feature is detected in >50% of experimental samples, it is removed from data analysis. Then the missing values of raw data were filled up by half of the minimum value. In addition, internal standard normalization method was employed in this data analysis. Finally, features with RSD >30% should be removed from the subsequent analysis. The resulted threedimensional data involving the peak number, sample name, and normalized peak area were fed to R package MetaboAnalystR for principal component analysis (PCA) and orthogonal partial least squares discriminant analysis (OPLS-DA). Principal component analysis (PCA) showed the distribution of origin data. In order to obtain a higher level of group separation and get a better understanding of variables responsible for classification, supervised orthogonal partial least squares discriminant analysis (OPLS-DA) were applied, and then to be calculate the value of R2 and Q2. R2 indicates how well the variation of a variable is explained and Q2 means how well a variable could be predicted. Afterwards, to check the robustness and predictive ability of the OPLS-DA model, 200 times permutation was further conducted. To refine this analysis, the first principal component of variable importance in the projection (VIP) was obtained. The VIP values summarize the contribution of each variable to the model. The metabolites with VIP >1 and *p* < 0.05 (student's test) and foldchange >1.5 or <0.67 were considered as significantly changed metabolites. In addition, commercial databases including KEGG (http://www.kegg.jp) and MetaboAnalyst (http://www.metaboanalyst.ca/) was utilized to search for the pathways of metabolites.

### Enzyme-linked immunosorbent assay

To validate therapeutic effects of aspirin and indobufen, plasma derived from five individuals in each group (CK, PTA_UN, PTA_A and PTA_INDO) was used for detection of inflammation-related factors (IL-1β, IL-6 and TNF-α) that were common biomarkers in the progression of vascular restenosis. ELISA kits for plasma IL-1β (ab214025), IL-6 (ab178013) and TNF-α (ab181421) were purchased from Abcam (Cambridge, UK). The optical density at 450 nm was measured using a SuperMax 3100 reader (Shanpu, Shanghai, China).

### Statistical analysis

Results are expressed as mean ± SEM or mean ± SD, as indicated. Statistical analyses were performed by one-way analysis of variance (ANOVA) with Tukey's test using the Graphpad Prism 8.0.2. Different lowercase letters indicated significant difference with *P* < 0.05 among groups.

## Discussion

Percutaneous Transluminal Angioplasty (PTA) is widely used in clinical practice to effectively ameliorate arterial stenosis or occlusion in patients with renal and cardiovascular diseases. However, due to irreversible mechanical injury to the vascular wall, patients often experience restenosis within six months post-operation, significantly impacting clinical outcomes (7). Although the molecular pathogenesis of post-PTA restenosis is unclear, potential causes include vascular inflammation, activation of platelet factors, and neointimal hyperplasia due to smooth muscle cell proliferation (26). Recently, research on blood metabolism has become crucial for the clinical diagnosis of vascular diseases and pathologies. Changes in plasma metabolite composition are vital indicators of disease onset, including compromised endothelial function, inflammatory reactions, oxidative stress, and atherosclerosis, all marked by specific alterations in blood metabolites (27). Hitherto, studies on blood metabolism during restenosis post-PTA remain scarce.

In this study, we used LC-MS/MS techniques to analyze blood metabolite alterations in patients post-PTA, aiming to identify aberrant metabolite expression patterns throughout their disease progression. Our data highlighted active choline and amino acid metabolism in patients post-PTA. Choline metabolism is closely correlated with vascular inflammation and atherosclerosis (28). For instance, cholesterol is a major component of atherosclerotic plaques. Elevated LDL-C levels in the blood promote atherosclerotic plaque formation and arterial wall damage, leading to vascular stenosis and thrombosis (29). Similarly, decreased cholinesterase activity, resulting in higher choline levels, exacerbates atherosclerosis (30). We observed significant decreases in various choline metabolism-related metabolites in patient plasma post-PTA, including cholesterol, 1-palmitoylysophosphatidylcholine, phosphocholine, SOPC, glycochenodeoxycholate, glycerophosphate, and glycerophosphocholine, even falling below levels in healthy individuals. Thus, monitoring blood choline metabolism changes can effectively gauge disease progression in patients with vascular restenosis. Additionally, amino acid metabolism in the blood influences protein synthesis, energy metabolism, metabolic regulation, and neurotransmitter synthesis. We found decreased levels of L-glutamate, L-isoleucine, L-valine, gamma-glutamylcysteine, and L-serine in patient plasma, serving as important biomarkers for kidney disease, metabolic disorders, and cardiovascular diseases (31). However, their reduced levels may lead to metabolic deficiencies, warranting further clinical research.

Aspirin and indobufen are crucial in treating vascular restenosis (13). Metabolomic analysis showed significant improvements in amino acid metabolism in patients treated with aspirin and indobufen post-PTA. Amino acid metabolites were present in several metabolic pathways, such as choline, sphingolipid, and caffeine metabolism. Arginine-related metabolites were frequently characterized. Arginine metabolism plays a key role in nitric oxide synthesis, affecting vasodilation, blood pressure regulation, neurotransmission, and immune regulation (32, 33). Since kidneys play a critical role in arginine metabolism, renal function significantly influences blood arginine levels. In individuals with kidney disease, a decreased renal glomerular filtration rate may elevate blood arginine levels (33). Consequently, arginine metabolism abnormalities may impair vascular dilation, increasing the risk of hypertension and other cardiovascular diseases. Our study found elevated levels of arginine-related metabolites, including argininosuccinic acid and L-citrulline, in the plasma of post-PTA patients, exceeding those in healthy individuals. Studies suggest hyperargininemia can impair neurological function and precipitate neurodegenerative diseases or neuropathies (34). In contrast, post-PTA patients on aspirin exhibited activation of 17 arginine metabolism-related metabolites, while those on indobufen showed activation of Endogenous levels in these patients approached those of healthy individuals, regulated by aspirin and indobufen. Clinically, indobufen serves as an alternative for patients intolerant to aspirin for gastrointestinal issues. Our findings suggest indobufen has a lesser impact on arginine metabolism regulation and activation, indicating a minimal overall effect on blood metabolism changes. Therefore, indobufen, affecting arginine metabolism homeostasis minimally, is more suitable for clinical use. However, indobufen negatively affects patients with a history of steatohepatitis. In conclusion, it is crucial to consider the overall patient condition and drug interactions when using these medications to prevent adverse reactions and complications.

Vascular restenosis in kidney disease patients can lead to various comorbidities, significantly burdening health. Restenosis-induced blood flow restriction increases vascular resistance, affecting blood pressure and glucose regulation, potentially leading to hypertension and diabetes (35). Abnormal blood metabolite levels are commonly used to assess various kidney, cardiovascular, and metabolic diseases (36). Therefore, monitoring blood metabolic changes in patients with hypertension and diabetes undergoing PTA is crucial. In summary, this study highlights the impact of aspirin and indobufen therapy post-PTA on blood metabolism homeostasis, identifying several promising biomarkers for diagnosing disease progression in patients with post-PTA restenosis.

## Conclusions

In summary, we provided the first global plasma metabolomic profile of vascular restenosis (VR) using LC-MS/MS sequencing. We identified argininosuccinic acid, pro-leu, L-citrulline, his-glu, 1-palmitoyllysophosphatidyl choline, phosphocholine and L-glutamate as important biomarkers for diagnosing VR symptoms. We identified 21 characteristic metabolites in vovled in arginine metabolism specifically linked to aspirin and indobufen, promising as early diagnostic biomarkers.

## Data Availability

The authors declare that all data supporting the findings of this study are available within the article. Any raw data can be obtained from the corresponding author upon request.
